# Association of Mitochondrial DNA Copy Number With Atopic Dermatitis in Children

**DOI:** 10.1002/clt2.70214

**Published:** 2026-10-12

**Authors:** Ju Chang‐Chien, Tzu‐Han Su, Xin‐Hui Lin, Yu‐Hsuan Liao, Yi‐Fen Tsai, Yin Szu, Yen‐Chieh Meng, Ming‐Ling Kuo, Hui‐Ju Tsai, Tsung‐Chieh Yao

**Affiliations:** ^1^ Division of Allergy, Asthma, and Rheumatology Department of Pediatrics Chang Gung Memorial Hospital Taoyuan Taiwan; ^2^ School of Medicine Chang Gung University College of Medicine Taoyuan Taiwan; ^3^ Department of Medical Education Chang Gung Memorial Hospital Taoyuan Taiwan; ^4^ Department of Public Health Kaohsiung Medical University Kaohsiung Taiwan; ^5^ Interdisciplinary Program of Life Science and Medicine National Tsing Hua University Hsinchu Taiwan; ^6^ Institute of Population Health Sciences National Health Research Institutes Zhunan Taiwan; ^7^ Department of Microbiology and Immunology Graduate Institute of Biomedical Sciences College of Medicine Chang Gung University Taoyuan Taiwan; ^8^ Department of Pediatrics New Taipei Municipal TuCheng Hospital New Taipei Taiwan; ^9^ Department of Medical Science National Tsing‐Hua University Hsinchu Taiwan

**Keywords:** atopic dermatitis, biomarker, children, mitochondrial DNA copy number

## Abstract

**Background:**

Mitochondrial DNA copy number (mtDNA‐CN), a marker associated with mitochondrial function and cellular health, has been implicated in several chronic inflammatory diseases. Atopic dermatitis is a common chronic inflammatory skin disorder, yet the relationship between mtDNA‐CN and childhood atopic dermatitis remains unclear. This study aimed to investigate the association between mtDNA‐CN and atopic dermatitis in a population‐based cohort of school‐aged children.

**Methods:**

We included 1329 singleton children (56.4% boys; mean age ± standard deviation: 6.5 ± 0.5 years) from the Longitudinal Investigation of Global Health in Taiwanese Schoolchildren (LIGHTS) cohort. Genomic DNA was extracted from peripheral blood, and mtDNA‐CN was quantified by real‐time quantitative polymerase chain reaction. Multivariable logistic regression models were applied.

**Results:**

Among the 1329 children, 328 (24.7%) children had physician‐diagnosed atopic dermatitis. Mean mtDNA‐CN was lower in children with atopic dermatitis than in those without (289.62 ± 102.06 vs. 299.47 ± 91.90; *p* = 0.03). Each one‐unit increase in log‐transformed mtDNA‐CN was associated with 28% lower odds of atopic dermatitis after adjustment for potential confounders (adjusted odds ratio: 0.72; 95% confidence interval: 0.54–0.96). Subgroup analyses showed that this inverse association was more pronounced among children breastfed for ≥ 3 months and those with a birth weight ≥ 2500 g.

**Conclusion:**

This study provides novel evidence of an inverse association between mtDNA‐CN and atopic dermatitis in children. Further longitudinal and mechanistic studies are warranted to confirm these findings and to evaluate the potential utility of mtDNA‐CN as a biomarker for atopic dermatitis.

## Graphical abstract

In a population‐based cohort of school‐aged children, higher mitochondrial DNA copy number was associated with lower odds of atopic dermatitis, with a more pronounced association among children breastfed for longer durations and those with a birth weight ≥ 2500 g. These findings suggest a potential role of mitochondrial biology in childhood atopic dermatitis.

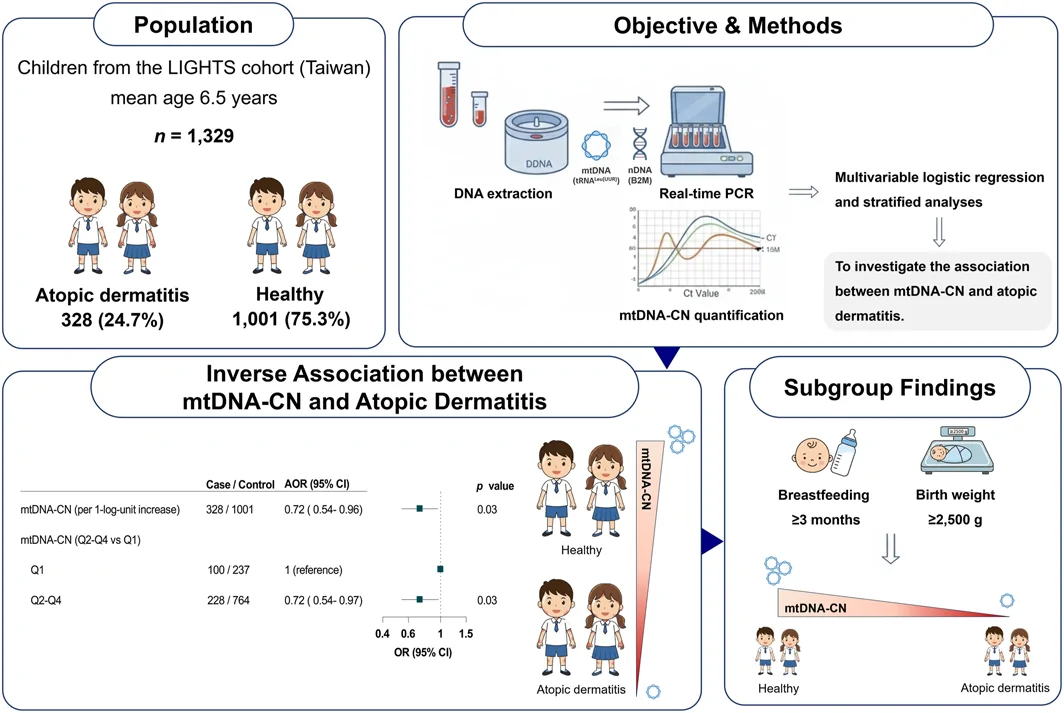

**Abbreviations**
AOR: adjusted odds ratio
ATP: adenosine triphosphate
BMI: body mass index
CGMH: Chang Gung Memorial Hospital
CIs: confidence intervals
Ct: cycle threshold
DAMP: damage‐associated molecular pattern
ETS: environmental tobacco smoke
ISAAC: International Study of Asthma and Allergies in Childhood
LIGHTS: Longitudinal Investigation of Global Health in Taiwanese Schoolchildren
mtDNA‐CN: mitochondrial DNA copy number
OXPHOS: oxidative phosphorylation
PM_2.5_
: particulate matter with a diameter ≤ 2.5 μm
qPCR: real‐time polymerase chain reaction
ROS: reactive oxygen species
SD: standard deviation
VIFs: variance inflation factors

## 1 Introduction

Atopic dermatitis is a common chronic inflammatory skin disease characterized by recurrent eczematous lesions and intense pruritus, often resulting in substantial impairment of quality of life [1, 2]. The prevalence of atopic dermatitis has increased markedly worldwide, including in Taiwan, making atopic dermatitis a growing public health challenge and economic burden [3, 4]. While genetic predisposition, immune dysregulation, and microbial imbalance are well‐recognized contributors to the pathogenesis of atopic dermatitis, recent evidence suggests that systemic metabolic disturbances and oxidative stress also play pivotal roles in the development of atopic dermatitis [5, 6, 7], raising the possibility that mitochondrial dysfunction may be involved in the complex pathophysiology of atopic dermatitis.

Mitochondria are essential organelles that generate adenosine triphosphate (ATP) and regulate oxidative metabolism as well as inflammatory signaling pathways [8, 9]. Mitochondrial dysfunction has been linked to several chronic inflammatory diseases [10]. However, reliable clinical indicators of mitochondrial dysfunction remain limited. Recent evidence suggests that mitochondrial DNA copy number (mtDNA‐CN) is a readily measurable and biologically informative indicator representing mitochondrial function [11]. Lower mtDNA‐CN has been observed in patients with several chronic inflammatory and metabolic disorders, compared with healthy controls [12]. Given that atopic dermatitis is one of the most common chronic inflammatory skin disorders [13], it is biologically plausible that mtDNA‐CN may contribute to or reflect the mitochondrial dysregulation underlying the disease.

Despite this biologic plausibility, evidence linking mtDNA‐CN to atopic dermatitis, particularly in children, is lacking. To address this knowledge gap, we investigated the association between mtDNA‐CN and physician‐diagnosed atopic dermatitis among school‐aged children participating in the Longitudinal Investigation of Global Health in Taiwanese Schoolchildren (LIGHTS) cohort.

## 2 Methods

### 2.1 Study Participants

This study included 1329 singleton children from the LIGHTS cohort, a prospective population‐based cohort designed to longitudinally evaluate the impacts of early‐life environmental exposures and genetic predisposition on health outcomes, particularly allergic diseases. Details of subject enrollment and data collection have been previously described [14, 15]. A flowchart of subject selection is shown in Figure 1. In brief, a total of 1513 children born at the Chang Gung Memorial Hospital (CGMH) between 2010 and 2011 were initially recruited. After excluding 162 multiple births and 22 children with missing data on atopic dermatitis (*n* = 9) or mtDNA‐CN (*n* = 13), 1329 singleton children were included in the subsequent analysis. Parents of the study children completed a modified International Study of Asthma and Allergies in Childhood (ISAAC) questionnaire [16], providing demographic information, environmental exposures, socioeconomic data, and physician‐diagnosed allergic diseases. Each child underwent a clinical evaluation by a physician. Perinatal information was retrieved from the electronic medical records in the CGMH. Peripheral blood samples for mtDNA‐CN measurement and assessment of atopic dermatitis status were obtained during the same follow‐up visit (mean age: 6.5 ± 0.5 years). This study was approved by the Institutional Review Board of Chang Gung Medical Foundation (No. 202201967B0). Written informed consent was obtained from parents or legal guardians of all subjects.

**FIGURE 1:**
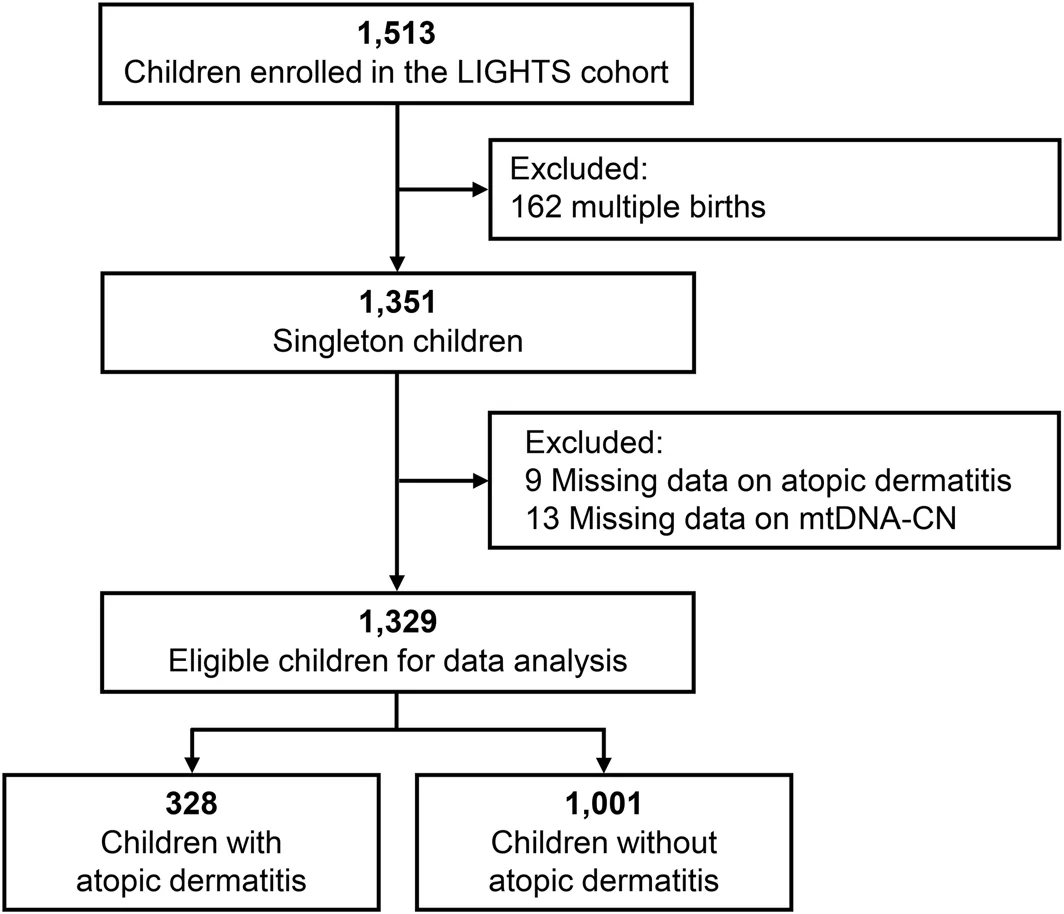
Flowchart of subject selection in the study cohort. LIGHTS, Longitudinal Investigation of Global Health in Taiwanese Schoolchildren; mtDNA‐CN, mitochondrial DNA copy number.

### 2.2 Measurement of Mitochondrial DNA Copy Number

The mtDNA‐CN in peripheral blood leukocytes was quantified using a quantitative real‐time polymerase chain reaction (qPCR) assay as previously described [17, 18, 19, 20]. Genomic DNA was isolated from peripheral blood cells using a commercial DNA extraction kit (RBC Bioscience). DNA quality was assessed by spectrophotometry, and only samples with an A260/A280 ratio between 1.8 and 2.0 were included. Each qPCR reaction contained 20 ng of genomic DNA. The mitochondrial gene *tRNA* ^*Leu(UUR)*^ and the single‐copy nuclear reference gene *B2M* were amplified using iQ SYBR Green Supermix on a Bio‐Rad CFX96 Real‐Time PCR Detection System. Thermal cycling consisted of an initial denaturation at 95°C for 10 minutes, followed by 40 PCR cycles of 95°C for 15 seconds and 62°C for 1 minute. Primer sequences are provided in Supporting Information S1: Table S1.

All samples were analyzed in triplicate. An intra‐assay coefficient of variation (CV) threshold of < 1.2% was applied, and samples exceeding this threshold were reanalyzed. To assess inter‐plate variation, calibrator DNA from K562 cells was included on every qPCR plate as an internal quality control. The inter‐assay CVs were 2.73% for *tRNA* ^*Leu(UUR)*^ and 1.10% for *B2M*. Laboratory personnel performing the qPCR assays were blinded to participants' atopic dermatitis status.

Relative mtDNA‐CN was calculated using the ΔCt method, where ΔCt = Ct(*B2M*)—Ct(*tRNA* ^*Leu(UUR)*^). In detail, relative mtDNA‐CN was expressed as 2 × 2^ΔCt^ in arbitrary relative units. The multiplication factor of 2 accounts for the diploid nuclear genome represented by *B2M*, thereby providing a relative estimate of mtDNA content normalized to nuclear DNA and approximating the mtDNA copy number per nucleated cell.

### 2.3 Definition of Atopic Dermatitis

Information on atopic dermatitis was obtained from a modified ISAAC questionnaire completed by the parents of participating children [16]. Children were defined as having atopic dermatitis if parents reported a physician‐diagnosed atopic dermatitis based on the question: “Has your child ever had atopic dermatitis diagnosed by a physician?” In addition, each child underwent a clinical evaluation by a physician to verify the reported diagnosis according to clinical diagnostic criteria [21].

### 2.4 Statistical Analysis

Categorical variables were compared using the chi‐squared test, while continuous variables were analyzed using Student's *t*‐test. Because mtDNA‐CN exhibited a right‐skewed distribution, the values were log‐transformed for regression analyses and additionally categorized into quartiles. For categorical analyses, the lowest quartile (Q1) served as the reference group, whereas the upper three quartiles (Q2–Q4) were combined because they showed a similar magnitude of association with atopic dermatitis. Associations between mtDNA‐CN and atopic dermatitis were examined using multivariable logistic regression models, adjusting for age, sex, body mass index (BMI), parental allergic diseases, annual household income (as a proxy of socioeconomic status), parental educational level, environmental exposures (e.g., environmental tobacco smoke [ETS] and 1‐year average particulate matter with a diameter ≤ 2.5 μm [PM_2.5_]), and perinatal factors (e.g., birth weight, breastfeeding and preterm birth). Adjusted odds ratios (AORs) and 95% confidence intervals (CIs) were reported, and the results were visualized using forest plots. The AORs represent the change in the odds of atopic dermatitis for each one‐unit increase in log‐transformed mtDNA‐CN. Stratified analyses were performed to assess potential effect modification by breastfeeding (< 3 months vs. ≥ 3 months) and birth weight (< 2500 g vs. ≥ 2500 g). We included product terms for mtDNA‐CN × breastfeeding and mtDNA‐CN × birth weight, separately, in the regression models to examine potential interactions. Model diagnostics were performed by assessing the linearity of log‐mtDNA‐CN in the logit using the Box‐Tidwell test, influential observations using Cook's distance, and multicollinearity using variance inflation factors (VIFs). The Box–Tidwell test indicated no violation of the linearity assumption (*p* = 0.46). No influential observations were identified based on Cook's distance, and VIFs ranged from 1.01 to 1.13, indicating no substantial multicollinearity. All data analyses were performed using Statistical Analysis System, version 9.4 (SAS Institute, Cary, NC, USA). A *p* value less than 0.05 was considered statistically significant.

## 3 Results

### 3.1 Subject Characteristics

Table 1 summarizes the demographic and clinical characteristics of the study subjects according to the presence or absence of atopic dermatitis. Among the 1329 singleton children, 328 (24.7%) children had physician‐diagnosed atopic dermatitis and 1001 (75.3%) children did not. The mean age of the study population was 6.5 years (standard deviation [SD]: 0.5) and 750 (56.4%) were male. Children with atopic dermatitis had significantly higher prevalences of asthma (19.5% vs. 12.4%; *p* = 0.002), allergic rhinitis (61.6% vs. 47.4%; *p* < 0.001), and parental allergic diseases (80.2% vs. 66.8%; *p* < 0.001) than those without atopic dermatitis (Table 1). No significant differences were observed in age, sex, BMI, birth weight, preterm birth, breastfeeding, parental educational level, ETS exposure, and season of sampling between the two groups. Mean mtDNA‐CN was lower in children with atopic dermatitis than in those without (289.62 ± 102.06 vs. 299.47 ± 91.90; *p* = 0.03).

**TABLE 1:** Demographic and clinical characteristics of 1329 singleton children with and without atopic dermatitis in the LIGHTS cohort.

Variable	With atopic dermatitis (*N* = 328)	Without atopic dermatitis (*N* = 1001)	*p* value^a^
Age, mean (SD), *y*	6.5 (0.5)	6.5 (0.5)	0.92
Sex, male, *n* (%)	194 (59.15)	556 (55.54)	0.25
Body mass index, kg/m^2^, mean (SD)	15.98 (2.60)	15.77 (2.33)	0.19
Birth weight ≥ 2500 (g), *n* (%)	291 (88.72)	863 (86.21)	0.24
Preterm birth, *n* (%)	45 (13.72)	160 (15.98)	0.32
Breastfeeding ≥ 3 months, *n* (%)	211 (64.53)	667 (66.63)	0.48
Asthma, *n* (%)	64 (19.5)	124 (12.4)	**0.002**
Allergic rhinitis, *n* (%)	202 (61.6)	474 (47.4)	**<** **0.001**
Parental allergic diseases, *n* (%)	263 (80.2)	668 (66.8)	**<** **0.001**
Asthma	40 (12.19)	107 (10.69)	0.51
Allergic rhinitis	230 (70.12)	606 (60.54)	**0.002**
Atopic dermatitis	142 (43.29)	199 (19.88)	**<** **0.001**
Parental educational level			0.17
Graduate school or above, *n* (%)	100 (30.49)	268 (26.77)	
University, *n* (%)	180 (54.88)	608 (60.74)	
High school or below, *n* (%)	48 (14.63)	125 (12.49)	
Environmental tobacco smoke exposure, *n* (%)	116 (35.4)	385 (38.5)	0.32
Season of sampling, *n* (%)			0.43
Spring (March to May), *n* (%)	86 (26.22)	269 (26.87)	
Summer (June to August), *n* (%)	69 (21.04)	249 (24.88)	
Autumn (September to November), *n* (%)	93 (28.35)	251 (25.07)	
Winter (December to February), *n* (%)	80 (24.39)	232 (23.18)	
Mitochondrial DNA copy number, mean (SD)	289.62 (102.06)	299.47 (91.90)	**0.03**

Abbreviations: LIGHTS, Longitudinal Investigation of Global Health in Taiwanese Schoolchildren; SD, standard deviation.

^a^ Bold values indicate statistical significance at *p* < 0.05.

### 3.2 Association of Mitochondrial DNA Copy Number With Atopic Dermatitis

Figure 2 shows the inverse association of mtDNA‐CN with atopic dermatitis in children. Specifically, each one‐unit increase in log‐transformed mtDNA‐CN was independently associated with 28% lower odds of atopic dermatitis after adjustment for age, sex, BMI, birth weight, preterm birth, breastfeeding, parental allergic diseases, parental educational level, annual household income, ETS exposure, and 1‐year average PM_2.5_ (AOR: 0.72; 95% CI: 0.54–0.96). When mtDNA‐CN was analyzed categorically, children in the upper three quartiles (Q2‐Q4) had 28% lower odds of atopic dermatitis than those in the lowest quartile (Q1) (AOR: 0.72; 95% CI: 0.54–0.97).

**FIGURE 2:**
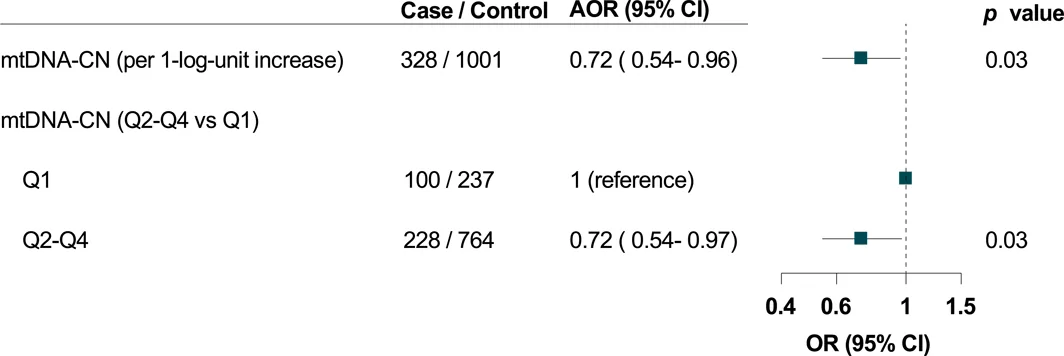
Association between mitochondrial DNA copy number and atopic dermatitis. AOR, adjusted odds ratio; CI, confidence interval; mtDNA‐CN, mitochondrial DNA copy number. AORs were obtained from multivariate logistic regression models adjusted for age, sex, body mass index, birth weight, preterm birth, breastfeeding, parental allergic diseases, parental educational level, annual household income, environmental tobacco smoke exposure, and 1‐year average PM_2.5_.

### 3.3 Stratified Analyses

We further examined the association between mtDNA‐CN and atopic dermatitis within subgroups classified by breastfeeding and birth weight. As shown in Figure 3, higher mtDNA‐CN was associated with a lower likelihood of atopic dermatitis among children who were breastfed for ≥ 3 months, when analyzed as a continuous variable (AOR: 0.70; 95% CI: 0.49–0.98) and as a categorical variable (AOR: 0.64; 95% CI: 0.45–0.93). In contrast, no significant association was observed among children who were breastfed for < 3 months. Similarly, in the analyses stratified by birth weight (Figure 4), an inverse association between mtDNA‐CN and atopic dermatitis was evident only among children with a birth weight ≥ 2500 g (AOR: 0.73; 95% CI: 0.54–0.99 for continuous analyses; AOR: 0.69; 95% CI: 0.51–0.95 for categorical analyses), whereas no significant association was found among those with a birth weight < 2500 g. Neither the interaction between mtDNA‐CN and breastfeeding (*p* = 0.64) nor that between mtDNA‐CN and birth weight (*p* = 0.86) was statistically significant.

**FIGURE 3:**
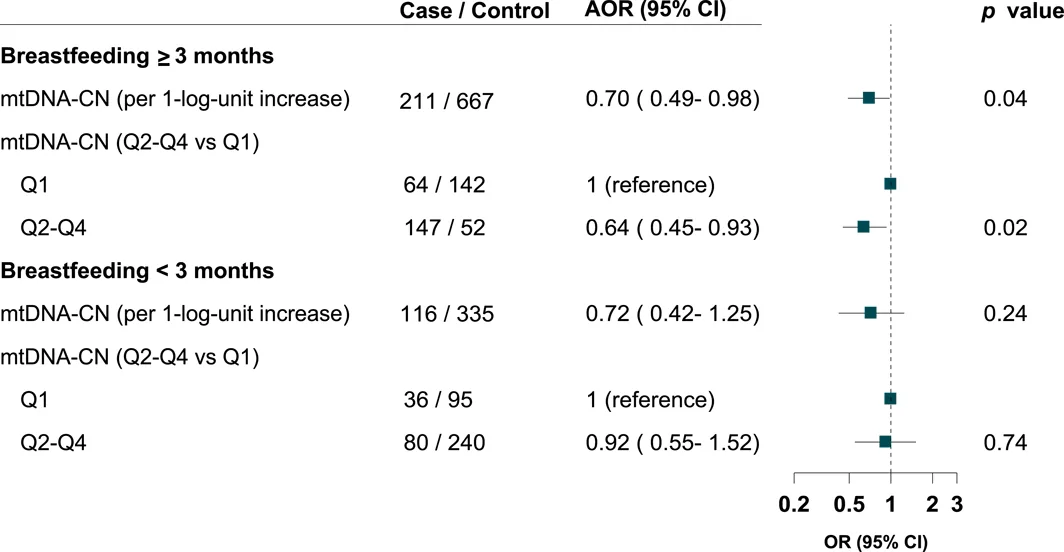
Association between mitochondrial DNA copy number and atopic dermatitis, stratified by breastfeeding. AOR, adjusted odds ratio; CI, confidence interval; mtDNA‐CN, mitochondrial DNA copy number. AORs were obtained from multivariate logistic regression models adjusted for age, sex, body mass index, birth weight, preterm birth, breastfeeding, parental allergic diseases, parental educational level, annual household income, environmental tobacco smoke exposure, and 1‐year average PM_2.5_.

**FIGURE 4:**
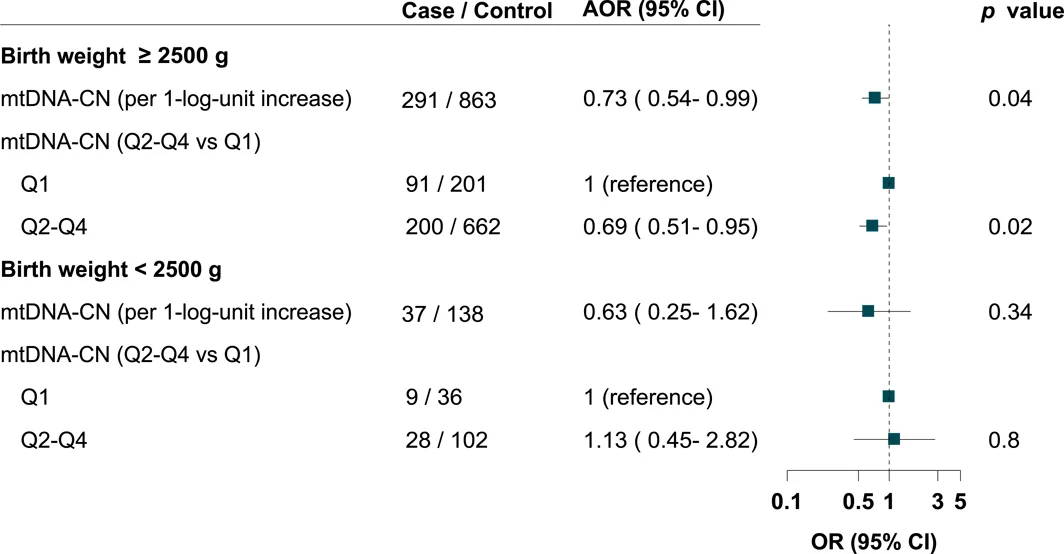
Association between mitochondrial DNA copy number and atopic dermatitis, stratified by birth weight. AOR, adjusted odds ratio; CI, confidence interval; mtDNA‐CN, mitochondrial DNA copy number. AORs were obtained from multivariate logistic regression models adjusted for age, sex, body mass index, birth weight, preterm birth, breastfeeding, parental allergic diseases, parental educational level, annual household income, environmental tobacco smoke exposure, and 1‐year average PM_2.5_.

## 4 Discussion

In this population‐based cohort study of 1329 school‐aged children, we observed a significant inverse association between mtDNA‐CN and atopic dermatitis. This association remained after adjustment for key confounders, including age, sex, BMI, birth weight, preterm birth, breastfeeding, parental allergic diseases, parental educational level, annual household income, ETS exposure, and 1‐year average PM_2.5_. Stratified analyses further indicated that the inverse association was more pronounced among children who were breastfed for ≥ 3 months, and those with a birth weight ≥ 2500 g. To the best of our knowledge, this is the first cohort study to demonstrate an association between mtDNA‐CN and atopic dermatitis in children. These findings provide novel evidence supporting an association between mtDNA‐CN and atopic dermatitis, suggesting a potential role of mitochondrial dysfunction in the early‐life pathogenesis of atopic dermatitis. Although the difference in mean mtDNA‐CN between children with and without atopic dermatitis was modest relative to the variability of the measure, the observed association may still be biologically relevant because mtDNA‐CN is a marker associated with mitochondrial function, and alterations in mtDNA‐CN have been linked to mitochondrial dysfunction and oxidative stress implicated in the pathogenesis of atopic dermatitis. However, the clinical utility of mtDNA‐CN as a standalone diagnostic or predictive biomarker remains unknown, and its potential value should be evaluated in combination with other biomarkers in future studies.

This cohort study provides novel evidence supporting an inverse association of mtDNA‐CN and atopic dermatitis in children. To our knowledge, no previous studies have directly examined this relationship. Nonetheless, our findings align with previous studies reporting inverse associations between mtDNA‐CN and other allergic or inflammatory diseases, including asthma, diabetes mellitus, and cardiovascular disease [11, 22, 23]. Collectively, these studies suggest that reduced mtDNA‐CN may reflect mitochondrial dysfunction, leading to impaired cellular resilience to oxidative and inflammatory stress and thereby increasing disease susceptibility, severity or exacerbation risk [11, 24, 25]. For example, Xu et al. reported that lower mtDNA‐CN was associated with a greater risk of asthma exacerbation [11], although another study did not find such a relationship [26]. Currently, the evidence regarding the relationship between mtDNA‐CN and allergic diseases remains inconclusive, as some studies have shown elevated mtDNA‐CN during the onset or active phase of asthma or allergic rhinitis [26, 27]. These discrepancies could explain in part that mtDNA‐CN levels may represent dynamic mitochondrial responses that vary with disease activity, inflammatory status, or acute exacerbations. Therefore, our findings should be interpreted with caution, and longitudinal studies are warranted to clarify the temporal and mechanistic nature of these associations. Interestingly, the inverse association between mtDNA‐CN and atopic dermatitis was observed only among children who were breastfed for ≥ 3 months or had a birth weight ≥ 2500 g. However, because neither interaction was statistically significant, these stratified findings should be interpreted with caution. Although the underlying mechanisms remain unclear, these observations raise the possibility that early‐life nutritional and developmental factors may modulate the association between mtDNA‐CN and atopic dermatitis in children. Further investigations are needed to confirm the subgroup findings and to elucidate their regulatory biological relevance.

Some plausible biological interpretations may underlie the observed association between mtDNA‐CN and atopic dermatitis. First, mtDNA‐CN can be influenced by genetic factors, oxidative stress, and environmental exposures. Such alterations may reflect mitochondrial dysfunction, which in turn may contribute to development of atopic dermatitis [12]. Secondly, because mtDNA encodes key components of the oxidative phosphorylation (OXPHOS) system, a higher mtDNA‐CN may indicate greater gene dosage and transcriptional potential for energy production [28, 29]. This speculative compensatory response could promote cellular recovery and mitigate inflammatory processes. Third, previous studies suggest that higher mtDNA‐CN helps maintain adequate mitochondrial capacity and antioxidant defenses, thereby stabilizing reactive oxygen species (ROS) levels and mitigating oxidative stress‐induced inflammation [28, 30, 31]. Furthermore, sufficient mitochondrial function and balanced ROS production may prevent the release of damage‐associated molecular patterns (DAMPs) that activate innate immune pathways [32, 33]. Of note, the above plausible biological mechanisms related to mtDNA‐CN remain speculative in the context of AD.

The present study has several notable strengths. First, we leveraged data collected from a large, prospective, and well‐characterized cohort of 1329 schoolchildren, in which mtDNA‐CN was objectively quantified using standardized laboratory methods. Second, outcome ascertainment for atopic dermatitis was based on the well‐validated ISAAC questionnaire and verified by physician clinical evaluation in the LIGHTS cohort, supporting the reliability of outcome ascertainment.

On the other hand, some limitations of this study should be acknowledged. First, although the LIGHTS cohort is prospective, the present analysis was cross‐sectional because both mtDNA‐CN and atopic dermatitis status were assessed at the same follow‐up visit. Therefore, temporality cannot be established, and reverse causation cannot be excluded, as systemic inflammation associated with atopic dermatitis may itself influence mtDNA‐CN. Consequently, causal inference is not warranted. Second, residual confounding arising from unmeasured factors, including diet, physical activity, infections, medication use, current atopic dermatitis treatment and coexisting atopic conditions, may have influenced the observed associations despite adjustment for several important covariates. In addition, leukocyte subtype composition was unavailable, and mtDNA‐CN was measured in peripheral blood leukocytes rather than the most relevant tissues or immune cell populations, which may not fully reflect mitochondrial function in tissues directly involved in atopic dermatitis. Because no functional mitochondrial measurements were performed, we were unable to directly assess mitochondrial function or verify the proposed biological mechanisms underlying the observed association. Information on current disease status, disease severity, and age at onset was unavailable in the present study, precluding evaluation of whether the observed associations differed according to the clinical characteristics of atopic dermatitis. Similarly, potential selection bias (e.g., single‐hospital cohort, participation and retention patterns) and information bias (e.g., parental recall of physician‐diagnosed atopic dermatitis, misclassification of mild atopic dermatitis), might attenuate or distort the observed associations. Third, we did not adjust for multiple testing because the primary hypothesis concerned the association between mtDNA‐CN and atopic dermatitis. The subgroup analyses according to birth weight and breastfeeding were exploratory and should therefore be interpreted with caution because of the potential for type I error. Fourth, the study population consisted exclusively of Asian schoolchildren recruited from a single‐hospital cohort, which may limit the generalizability of our findings to other ethnicities or age groups.

In conclusion, this study provides novel evidence of an inverse association between mtDNA‐CN and atopic dermatitis in school‐aged children. The inverse association was more pronounced among children who were breastfed for ≥ 3 months and those with a birth weight ≥ 2500 g, although these subgroup findings should be interpreted cautiously given the absence of significant interaction effects. Further longitudinal and mechanistic studies are warranted to confirm these findings and to evaluate the potential utility of mtDNA‐CN as a biomarker for atopic dermatitis.

### 4.1 Key Message

The role of mitochondrial function in childhood atopic dermatitis remains poorly understood. In this population‐based cohort of school‐aged children, higher mitochondrial DNA copy number was independently associated with lower odds of atopic dermatitis after adjustment for major confounders, with the association being more pronounced among children breastfed for longer durations and those with a birth weight ≥ 2500 g. These findings suggest a potential role of mitochondrial biology in childhood atopic dermatitis.

## Author Contributions

**Ju Chang‐Chien:** writing – original draft, methodology, investigation. **Tzu‐Han Su:** writing – original draft, investigation. **Xin‐Hui Lin:** writing – original draft, formal analysis. **Yu‐Hsuan Liao:** writing – original draft. **Yi‐Fen Tsai:** formal analysis. **Yin Szu:** data curation, formal analysis, investigation. **Yen‐Chieh Meng:** investigation. **Ming‐Ling Kuo:** writing – review and editing. **Hui‐Ju Tsai:** writing – original draft, writing – review and editing, funding acquisition, supervision. **Tsung‐Chieh Yao:** conceptualization, writing – review and editing, funding acquisition, supervision.

## Funding

This work was supported by National Science and Technology Council of Taiwan (PI: Yao, NSTC 115‐2314‐B‐182‐034‐MY3 and NSTC 114‐2321‐B‐182‐005; and Tsai NSTC 111‐2314‐B‐400‐040‐MY3), Chang Gung Medical Foundation (PI: Yao, CMPRG3M1381‐3, CMRPG3L1111‐3, and OMRPG3Q0031), and National Health Research Institutes, Taiwan (PI: Tsai, PH‐114‐PP‐08).

## Conflicts of Interest

The authors declare no conflicts of interest.

## Supporting information


Supporting Information S1

## Data Availability

The data that support the findings of this study are available from the corresponding author upon reasonable request.
