# Supplementary material for: Association of Mitochondrial DNA Copy Number With Atopic Dermatitis in Children
Source: Clin Transl Allergy. 2026 Oct 12;16(10):e70214. doi: 10.1002/clt2.70214 (PMC13657800; doi:10.1002/clt2.70214)
Supplement: Supplementary file 1 [file CLT2-16-e70214-s001.docx]

**Supporting Information**

**Table S1.** Primers used for mitochondrial DNA copy number.

| **Target Gene** | **Forward Primer Sequence (5’-3’)** | **Reverse Primer Sequence (5’-3’)** |
| --- | --- | --- |
| *tRNA^Leu(UUR)^* | CACCCAAgAACAgggTTTgT | TggCCATgggTATgTTgTTA |
| *B2M* | TgCTgTCTCCATgTTTgATgTATCT | TCTCTgCTCCCCACCTCTAAgT |

**Table S2.** Demographic and clinical characteristics of 1,329 singleton children in the LIGHTS cohort according to quartiles of mitochondrial DNA copy number.

|  | **Mitochondrial DNA copy number** | | | |  |
| --- | --- | --- | --- | --- | --- |
| **Variable** | **Q1 (≤247)** | **Q2 (>247 ~ ≤294)** | **Q3 (>294 ~ ≤347)** | **Q4 (>347)** | ***p* value**^a^ |
| Age, mean (SD), y | 6.35 (0.50) | 6.42 (0.51) | 6.54 (0.52) | 6.56 (0.53) | **<0.001** |
| Sex, male, n (%) | 200 (59.35) | 182 (55.49) | 188 (55.79) | 180 (55.005) | 0.66 |
| Body mass index, kg/m², mean (SD) | 15.71 (2.34) | 15.77 (2.45) | 15.79 (2.31) | 16.04 (2.49) | 0.23 |
| Birth weight ≥2500 (g), n (%) | 292 (86.65) | 278 (84.76) | 293 (86.94) | 291 (88.99) | 0.46 |
| Preterm birth, n (%) | 45 (13.35) | 53 (16.16) | 59 (17.51) | 48 (14.68) | 0.47 |
| Breastfeeding ≥3 months, n (%) | 206 (61.13) | 201 (61.47) | 239 (70.92) | 232 (70.95) | **0.003** |
| Asthma, n (%) | 38 (11.31) | 44 (13.54) | 52 (15.52) | 54 (16.62) | 0.22 |
| Allergic rhinitis, n (%) | 176 (52.23) | 162 (49.69) | 168 (50.15) | 170 (51.99) | 0.88 |
| Parental allergic diseases, n (%) |  |  |  |  |  |
| Asthma | 40 (11.94) | 26 (8.05) | 34 (10.24) | 47 (14.73) | 0.05 |
| Allergic rhinitis | 217 (64.39) | 200 (61.35) | 218 (64.88) | 201 (62.23) | 0.74 |
| Atopic dermatitis | 87 (25.89) | 85 (26.40) | 71 (21.26) | 98 (30.72) | 0.05 |
| Parental educational level |  |  |  |  | 0.31 |
| Graduate school or above, n (%) | 101 (29.97) | 88 (26.83) | 91 (27.00) | 88 (26.91) |  |
| University, n (%) | 195 (57.86) | 190 (57.93) | 213 (63.20) | 190 (58.10) |  |
| High school or below, n (%) | 41 (12.17) | 50 (15.24) | 33 (9.79) | 49 (14.98) |  |
| Environmental tobacco smoke exposure, n (%) | 131 (38.87) | 130 (39.63) | 128 (37.98) | 112 (34.25) | 0.50 |
| Season of sampling, n (%) |  |  |  |  | **<0.001** |
| Spring (March to May), n (%) | 54 (16.02) | 89 (27.13) | 100 (29.67) | 112 (34.25) |  |
| Summer (June to August), n (%) | 108 (32.05) | 74 (22.56) | 58 (17.21) | 78 (23.85) |  |
| Autumn (September to November), n (%) | 106 (31.45) | 92 (28.05) | 87 (25.82) | 59 (18.04) |  |
| Winter (December to February), n (%) | 69 (20.47) | 73 (22.26) | 92 (27.30) | 78 (23.85) |  |
| Mitochondrial DNA copy number, mean (SD) | 189.71 (61.72) | 271.61 (13.83) | 320.26 (15.40) | 409.23 (79.52) | **<0.001** |

Abbreviation: **LIGHTS**: Longitudinal Investigation of Global Health in Taiwanese Schoolchildren; **SD**: standard deviation.

^a^Bold values indicate statistical significance at *p* < 0.05.
